# Large Genomes Are Associated With Greater Cell Size and Ecological Shift Towards More Nitrogen‐Rich and Higher‐Latitude Environments in Microalgae of the Genus *Synura*

**DOI:** 10.1111/jeu.70026

**Published:** 2025-07-02

**Authors:** Dora Čertnerová, Pavel Škaloud, Iva Jadrná, Martin Čertner

**Affiliations:** ^1^ Department of Botany Faculty of Science, Charles University Prague Czech Republic; ^2^ Research Department for Limnology University of Innsbruck Mondsee Austria; ^3^ Institute of Botany of the Czech Academy of Sciences Průhonice Czech Republic

**Keywords:** ecological requirements, evolution, flow cytometry, GC content, genome size, PGLS regression, silica scales

## Abstract

The nuclear genome is essential for encoding most of the genes required for cellular processes, but its size alone can alter the characteristics of cells and organisms. Yet, genome size variation and its ecological and evolutionary impacts, particularly in microorganisms, are not well understood. We used flow cytometry to estimate genome size and GC content in 53 evolutionary lineages of the microalgal genus *Synura* (Chrysophyceae, Stramenopiles). Genome size evolution was reconstructed in a phylogenetic framework using molecular markers. A set of genomic, morphological, and ecogeographic variables characterizing *Synura* lineages was evaluated and tested as predictors of genome size variation in phylogeny‐corrected statistical models. Both genome size and GC content varied widely in *Synura*, ranging from 0.19 to 3.70 pg of DNA and 34.0% to 49.3%, respectively. Genome size variation was mainly associated with cell size, less with silica scale size, and not with scale ultrastructure. Higher soil nitrogen, higher latitudes, and lower temperatures correlated with larger genomes. Genome size evolution in *Synura* shows potential dynamism, with increases confined to short terminal branches, indicating lower macroevolutionary stability. Lineages with larger genomes exhibited a narrower range of suitable ecological conditions, possibly due to selection acting deleteriously against larger genomes (and cells).

## 1 Introduction

Although qualitative differences in nuclear DNA, expressed by its nucleotide sequences, have been the focus of biologists for over half a century, the quantity of DNA per cell has received significantly less attention. The genome size and its variations remain largely unexplored, yet this knowledge may provide insight into key cellular, physiological, and ecological interactions. While genome size varies nearly 120,000‐fold among eukaryotes (Veldhuis et al. 1997; Corradi et al. 2010), this variation does not reflect the biological complexity of organisms (Gregory 2005). The amount of nuclear DNA, however, determines nucleus size (although some other mechanisms might be involved in controlling the nuclear size; Cantwell and Nurse 2019) and this also affects the size of the cell (Bennett 1971; Cavalier‐Smith 1978). The genome size‐cell size correlation, also known as the karyoplasmic ratio (Wilson 1925), may have further consequences for metabolic rates, the duration of mitosis and meiosis, or generation time (Vinogradov and Anatskaya 2006; Šímová and Herben 2012). The cumulative effect of these associations could be reflected in the physiology or ecology of organisms (Bennett 1987; Cavalier‐Smith 1982; Herben et al. 2012; Roberts et al. 2024; Roddy et al. 2020). As an example, the genome size could impact tolerance to stressful environmental conditions (Nardon et al. 2005; Šmarda et al. 2023), ecological niche breadth (Pyšek et al. 2018), or even speciation and diversification rates (Igea et al. 2017; Bhadra et al. 2023).

Most of the evidence for genome size correlations comes from plant and animal studies. These organisms are, however, complex multicellular entities where the direct effect of genome size on cells may be buffered at the level of tissues or entire bodies (e.g., lower number of cells compensating for their greater size). In addition, any genome size–phenotype associations may be further obscured by somatic tissue endopolyploidy, occurring in most multicellular organisms (Leitch and Dodsworth 2017; Neiman et al. 2017).

In contrast, unicellular eukaryotes (protists) provide a more straightforward model for studying the evolution of genome size and its consequences for species morphology and ecophysiology. As the cell represents the entire body and it is in direct contact with the environment, the phenotypic consequences are expected to be even more pronounced. It has been demonstrated that cell size (usually expressed as cell biovolume) can directly affect ecology in protists, as cell size is reflected in, for example, their photosynthetic activity, predator spectrum, or sinking rate (Garcia‐Pichel 1994; Finkel et al. 2001; Smetacek et al. 2004; Irwin et al. 2006). Interestingly, according to the “temperature‐size rule” (Montagnes and Franklin 2001; Atkinson et al. 2003) studied in amoebae, ciliates, diatoms, dinoflagellates, and various flagellates, the size of their cells decreases by 2.5% with every 1°C increase in environmental temperature. Because cell size is usually tightly linked to genome size, temperature optima may differ among species in association with their genome size. The genome size could thus serve as an ecological and evolutionary constraint, delimiting large‐scale species distributions in protists, or even be subject to natural selection (via cell size; Cavalier‐Smith 2005). Although the correlation between genome size and cell size has been shown repeatedly in protists (e.g., chrysophytes, diatoms or dinoflagellates; Connolly et al. 2008; von Dassow et al. 2008; Čertnerová and Škaloud 2020; LaJeunesse et al. 2005), there is a dearth of data exploring further ecological and evolutionary ramifications of this relationship (but see Roberts et al. 2024).

We chose representatives of the genus *Synura* as a model group to study the evolutionary consequences of genome size variation in protists. Although they are colonial flagellates, the individual cells often detach from the colony (especially during cultivation) and can therefore be easily studied separately. The genus *Synura* belongs to the chrysophytes (Chrysophyceae, Stramenopiles), also known as golden‐brown algae due to the presence of photosynthetic pigment fucoxanthin, which gives them their brownish color (Jeffrey et al. 2011). *Synura* species are among the most abundant microalgae in oligotrophic lakes, and some species are known to form blooms that can cause an unpleasant fishy odor in drinking water reservoirs (Nicholls and Gerrath 1985). Members of the genus *Synura* incorporate silicic acid, forming siliceous scales on their plasma membrane. Despite the enormous variability in architectural design, the overall morphology of scales remains species‐specific. It has been demonstrated, however, that the scale morphology can be somewhat shaped by environmental conditions (Nemcova et al. 2010; Pichrtová and Němcová 2011). As the scales are close‐fitting and precisely arranged to form a highly organized cell envelope, the question arises as to whether changes in cell size (often linked to changes in genome size) may have an effect on scale morphology in this group. A recent discovery shows that at least the size of scales and cells seems to be associated (Siver 2022). The genome size in *Synura* was so far almost exclusively studied on the species *S*. *petersenii* (but see Olefeld et al. 2018). Substantial intraspecific genome size variation was observed in *S*. *petersenii* during our previous study, though no clear associations between genome size and ecogeographical variables were detected (Čertnerová and Škaloud 2020). Therefore, we have shifted our focus to the level of the entire genus *Synura*, represented here by 53 evolutionary lineages, and using the values of 40 genomic, morphological, and environmental variables, we aim to explore the patterns of genome size variation and identify its potential correlates.

By applying phylogeny‐corrected statistical models, we address the following questions: (1) What is the extent of genome size variation within the genus *Synura* and does the distribution of this variation exhibit strong phylogenetic patterns? (2) Was the evolution of genome size closely linked to adjustments in cell size and, consequently, to the size and ornamentation of the silica scales covering the cell surface? (3) Could the variation in genome size be explained by other genomic (GC content) or environmental parameters? (4) Is the genome size associated with environmental variables?

## 2 Materials and Methods

### 2.1 Origin and Cultivation of the Investigated Strains

To establish new cultures for this study, water samples were taken using a 25‐μm mesh plankton net, and single cells or colonies were captured by micro‐pipetting and transferred into separate culture wells filled either with MES buffered DY‐IV (in case of *S. rubra* , *S. sphagnicola* and *S. synuroidea*; Andersen et al. 1997) or with WC medium (Guillard and Lorenzen 1972). The sampling details for new strains are listed in Table S1. All cultures were maintained at 17°C (cooling box Pol‐Eko Aparatura Sp.J., model ST 1, Wodzisław Śląski, Poland) with a 24‐h light mode under illumination of 30 μmol m^−2^ s^−1^ (TLD 18 W/33 fluorescent lamps, Philips, Amsterdam, Netherlands). Subsequently, the strains were transferred into Erlenmeyer flasks filled with 30 mL of growth medium and kept for longer cultivation, with re‐inoculations into a fresh medium every three months. To broaden our dataset, we supplemented it with data for 184 additional *Synura* strains (mainly strains of *S*. *petersenii*) from our previous studies, where the same methodology was applied (Čertnerová and Škaloud 2020; Čertnerová et al. 2022).

### 2.2 Phylogenetic Analyses

First, to determine species, nuclear ITS rDNA (ITS1, 5.8S, and ITS2 rDNA) was sequenced for new *Synura* isolates. Then, to fit models of trait evolution and conduct statistical analyses in a phylogenetic context, we constructed a phylogenetic tree of each unique nu ITS rDNA lineage based on two additional molecular markers, nuclear SSU rDNA and plastid *rbc*L.

To sequence these molecular markers, genomic DNA was extracted from a centrifuged pellet of cells by InstaGene Matrix (Bio‐Rad, Hercules, CA, USA), and the resulting supernatant was directly used as a PCR template. Amplifications of nu ITS rDNA were performed using primers Chryso_ITS_F and Chryso_ITS_R (Jadrná et al. 2021). When amplifying the nu SSU rDNA, we employed primers 18SF or 528F and 18SR (Katana et al. 2001; Montresor et al. 2004). The pt. *rbc*L was amplified using rbcL‐Chrys‐F1 or rbcL‐Chrys‐F2 and rbcL‐Chrys‐R or rbcL_R3 primers (Jo et al. 2011; Škaloudová and Škaloud 2013). All PCRs were carried out in a total volume of 10 μL with a PCR mix containing 0.1 μL of MyTaq DNA polymerase (Bioline, Memphis, TN, USA), 2 μL of MyTaq buffer (Bioline), 0.1 μL of each primer, 6.7 μL of double distilled water, and 1 μL of template DNA (not quantified). Amplifications were performed in Eppendorf Mastercycler ep Gradient 5341 (Eppendorf GmbH, Hamburg, Germany) using the following programs: 1 min of denaturation at 95°C for all gene regions, followed by 35 cycles of denaturation at 95°C (15 to 30 s), annealing at 52°C/50°C/40°C for nu ITS rDNA, nu SSU rDNA, and pt. *rbc*L, respectively (1 min) and elongation at 72°C (40 s for nu ITS rDNA and 1 min for nu SSU rDNA and pt. *rbc*L), concluded with a final extension at 72°C (7 min for nu ITS rDNA and 10 min for nu SSU rDNA and pt. *rbc*L) and held at 10°C. The PCR products were sized on a 0.8% agarose gel and then purified using MagJET Magnetic Bead‐based Nucleic Acid Purification (Thermo‐Fisher Scientific, Waltham, MA, USA). The purified DNA templates were sequenced using the Sanger sequencing method at Macrogen Inc. (Amsterdam, the Netherlands, https://www.macrogen‐europe.com).

New sequences were manually checked using SeqAssem ver. 9 (Hepperle 2004). The dataset was supplemented with GenBank‐extracted sequences and sequences used in our previous studies (Čertnerová et al. 2022; Škaloud et al. 2023). A nu SSU rDNA and pt. *rbc*L alignment was built for the following analyses using MEGA5 (Tamura et al. 2011) and comprised 55 *Synura* strains (belonging to each unique nu ITS rDNA lineage) and seven *Mallomonas* strains used as the outgroup (see Table S2).

To create a time‐calibrated phylogenetic tree, first, individual SSU rDNA and *rbc*L phylogenetic trees were inferred by the maximum likelihood (ML) analysis using RAxML ver. 8.1.20 (Stamatakis 2014) with 20 replicates, applying the GTRGAMMA evolutionary model and rapid bootstrapping as implemented in the CIPRES Science Gateway (Miller et al. 2010). The *rbc*L phylogenetic analysis was based on the codon‐partitioned dataset, with saturated nucleotide positions removed by a modified site‐stripping approach described in Škaloud, Kristiansen, and Škaloudová (2013). Since the topologies were highly congruent, we performed the final phylogenetic analysis based on the concatenated and partitioned alignment, using the Bayesian framework in BEAST ver. 1.10.4 (Suchard et al. 2018). Lognormal relaxed clock models were applied for the partitions, and a birth–death diversification process was selected as a prior on the distribution of node heights. For temporal calibration of the phylogeny, we used the time constraints of five selected nodes following the time‐calibrated phylogeny published by Jadrná et al. (2021): (1) the lineage consisting of first *Synura* representatives (110.6 Mya), (2) the stem of all Curtispinae taxa (92.5 Mya), (3) the stem of all Petersenianae taxa (38.1 Mya), (4) the lineage of *S. curtispina* and *S. spinosa* (54.5 Mya), (5) the lineage of *S*. *leptorrhabda*, *S*. *mamillosa*, and *S. echinulata* (23.4 Mya). The standard deviation of all constraints was set to 1.0. Three MCMC analyses were run for 50 million generations, sampling every 50,000 generations after 5 million generations removed as a burn‐in. We checked the parameter‐estimated convergence with Tracer ver. 1.7.1 (Rambaut et al. 2018) and then constructed the final chronogram with age estimation for all nodes. Trees were visualized using FigTree ver.1.4.2. (Rambaut 2016).

### 2.3 Estimation of Genome Size and DNA Base Content

To estimate genome size and GC content of the studied strains, we employed flow cytometry (FCM). Two fluorescent dyes, the base nonspecific, intercalating propidium iodide (PI) and the AT‐selective 4′,6‐diamidino‐2‐phenylindole (DAPI), were used separately to determine genome size and base content, respectively. Approximately two weeks before the planned FCM analyses, cultures were inoculated into fresh medium. For sample preparation, 1 mL of well‐grown culture was centrifuged (5 min, 2040× *g*; Eppendorf) and the superfluous medium was removed by pipetting. Consequently, 350 μL of ice‐cold nuclei isolation buffer Otto I (0.1 M citric acid, 0.5% Tween 20; Otto 1990) was added to the algal pellet, causing an osmotic rupture of cells and the release of the sample nuclei. The resulting suspension was thoroughly shaken and kept on ice. The plants *Bellis perennis* , wild clone (2C = 3.38 pg.; Schönswetter et al. 2007), *Carex acutiformis* , wild clone (2C = 0.82 pg.; Lipnerová et al. 2013), *Pisum sativum* cv. Ctirad (2C = 8.018 pg.; Šmarda et al. 2014), or *Solanum pseudocapsicum* , commercial clone (2C = 2.59 pg.; Temsch et al. 2010) were used as a (pseudo‐)internal standard, depending on the sample genome size. To release nuclei of the standard, a 20‐mg piece of fresh leaf tissue was chopped with a razor blade in a plastic Petri dish with 250 μL of ice‐cold Otto I buffer. Both suspensions (with algal and standard nuclei) were thoroughly mixed and filtered through a 42 μm nylon mesh into a special 3.5‐mL cuvette for direct use with the flow cytometer. Following a 20‐min incubation at room temperature, the sample was mixed with 1 mL of staining solution consisting of Otto II buffer (0.4 M Na_2_HPO_4_·12H_2_O; Otto 1990), 2 μL mL^−1^ β‐mercaptoethanol and either 50 μg mL^−1^ PI and 50 μg mL^−1^ RNase IIA or 4 μg mL^−1^ DAPI. To estimate genome size, the PI‐stained samples were analyzed using a Partec CyFlow SL cytometer (Partec GmbH, Münster, Germany) equipped with a green solid‐state laser (Cobolt Samba, 532 nm, 100 mW). For assessing GC content, the genome size measurements were supplemented with DNA content analysis on the DAPI‐stained samples using Partec PA II flow cytometer (Partec GmbH, Münster, Germany) equipped with a 488‐nm UV LED as a source of excitation light. During each analysis, 5000 nuclei were measured and the resulting FCM histograms were analyzed using FloMax ver. 2.4d (Partec). The first sample peak in the FCM histogram was identified as G_1_ (vegetative cells) and a second peak with twice the relative fluorescence as G_2_ (dividing cells) when present. The genome size (2C‐value) was calculated as sample G_1_ peak mean fluorescence/standard G_1_ peak mean fluorescence × standard 2C DNA content (according to Doležel 2005). Computation of the GC base content was done according to (Šmarda et al. 2008) via a publicly available Excel spreadsheet (http://sci.muni.cz/botany/systemgr/download/Festuca/ATGCFlow.xls). To minimize the effect of random instrumental shift, each strain was analyzed at least three times on separate days and the estimates averaged (except the strain CZ10D analyzed only once). We have previously shown that chrysophytes have an isomorphic haploid‐diploid life cycle and that the diploid life stage seems to predominate in cultivation (under the same setting as applied here; [Čertnerová et al. 2022]). Therefore, if only a single life‐cycle stage was detected within a species, we presumed that the strains were diploids (in case of *S. borealis* strain W76 and strains of *S*. *hibernica* and *S. sphagnicola* ).

### 2.4 Morphological Analysis

**FIGURE 1:**
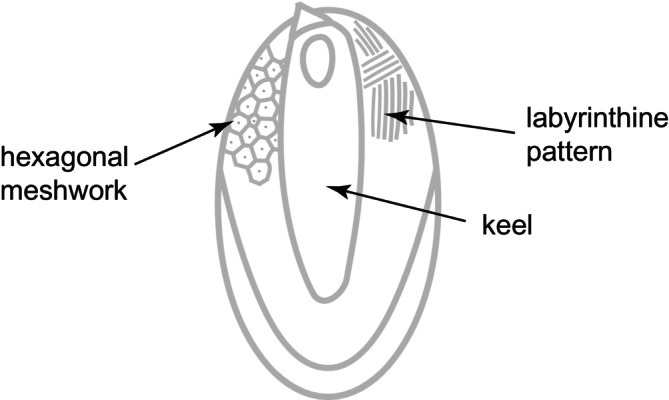
Schematic depiction of the siliceous body scale of *Synura* showing the morphological features analyzed for association with genome size.

Prior to scoring morphological traits, 50 μL of each strain at the exponential phase of growth was inoculated into 4 mL of fresh medium and cultivated for 2 weeks. After this period, microphotographs of individual cells were taken using a Leica DM2500 LED optical microscope with 40× magnification. The silica body scales were photographed using a transmission electron microscope (TEM) Jeol 1011 with integrated CCD camera Velvet (Olympus Soft Imaging Solution GmbH, Münster, Germany). The cell and scale sizes were later estimated for each strain using ImageJ ver. 1.45 s (Schneider et al. 2012) as object areas on the microphotograph. A presence of the most pronounced microstructures on scales (i.e., keel, hexagonal meshwork and labyrinthine pattern; Figure 1) and the scale length were either directly scored from microphotographs or measured using ImageJ, respectively. The final values of quantitative traits were derived as a median value of either 30 cells or scales measured per each strain (Table S3).

### 2.5 Ecogeographical Variables

In order to assess putative ecogeographical trends in the distribution of genome size diversity, we tested for associations between the genome size of *Synura* and environmental variables either directly scored at their original sites or extracted from publicly available databases. The populations of *Synura* spp. were sampled at 178 sites located across the Northern hemisphere (see Table S1), georeferenced and supplemented with three in situ measured parameters (i.e., water temperature, pH and conductivity). We downloaded 19 Bioclim variables of the WorldClim database ver. 2 (https://worldclim.org; Fick and Hijmans 2017) in the highest available resolution (30 arc sec = approximately 1 km^2^) and used ArcGIS 10.0 (ESRI, Redlands, CA, USA) to extract climate data for the sampling sites. We used tools available within the R packages “soilDB” v. 2.7.7 and “aqp” v. 1.42 to download soil data from the SoilGrids database (https://soilgrids.org; Hengl et al. 2017) in the highest resolution (250 m) and extract the values of 9 soil horizon variables for the sampling sites, respectively (Beaudette et al. 2013; Poggio et al. 2021). For each soil variable, the available data from all six soil horizons were averaged. Prior to statistical analysis, database‐derived data were checked for strong correlation among variables (*r* ≥ 0.85) and only one randomly selected variable from each pair of strongly correlated variables was retained. The reduced dataset consisted of 8 climatic and 9 soil variables: annual mean temperature (bio1), mean diurnal range (bio2), maximal temperature of warmest month (bio5), mean temperature of wettest quarter (bio8), mean temperature of driest quarter (bio9), annual precipitation (bio12), precipitation seasonality (bio15), precipitation of warmest quarter (bio18), bulk density of the fine earth fraction (bdod), cation exchange capacity of the soil (cec), volumetric fraction of coarse fragments (cfvo), proportion of clay particles in the fine earth fraction (clay), total soil nitrogen (nitrogen), soil pH (phh2o), proportion of sand particles in the fine earth fraction (sand), proportion of silt particles in the fine earth fraction (silt), soil organic carbon content in the fine earth fraction (soc).

### 2.6 Phylogeny‐Based Statistical Analyses

Due to the lack of independence expected among observations coming from within the same genus, we employed phylogeny‐corrected models for statistical data analysis. The values of genomic, morphological and ecological variables were averaged across all strains possessing an identical nu ITS rDNA sequence, and the final dataset thus consisted of 53 evolutionary lineages of *Synura*, each characterized by a set of 29 variables. This approach accounted for the unequal representation of lineages among the sampled strains (i.e., a natural consequence of blind in situ sampling) and eased combining the data with our previously published datasets, where the same methodology was applied (Čertnerová and Škaloud 2020; Čertnerová et al. 2022). Unless stated otherwise, all analyses were conducted in R v. 4.3.1 (R Core Team 2022). Ancestral states for genome size and cell size were reconstructed using the “fastAnc” function (fast ML estimation) and visualized on the tree with the “contMap” function, both from the R package “phytools” v. 0.6–60 (Revell 2012). The overall effect of phylogeny on variation in genome size (and its predictors) among *Synura* lineages was estimated using the branch length scaling parameter lambda (*λ*; Pagel 1999). As a measure of the strength of the phylogenetic signal, *λ* ranges from 0 to 1, corresponding to two extreme states in which either phylogeny has no effect on the data or the variation can be explained solely by the phylogenetic relationships among species, respectively.

The phylogenetic generalized least‐squares approach (PGLS), as implemented in the R package “caper” v. 1.0.1 (Orme et al. 2018), was used to conduct simple PGLS models separately for each of 28 genome size predictors (i.e., genomic, morphological and ecogeographic variables). Genome size was the response variable in all models, the ML method was used to estimate *λ*, while the values of two other scaling parameters (delta and kappa) were fixed to one. Some of the variables were log‐transformed (GC content, water temperature, conductivity, cell size, scale size, scale length, bio05, bio12, cec, nitrogen, phh2o, and soc) or square root‐transformed (cfvo and sand) to improve their normality. For comparative purposes, the phylogeny‐corrected simple PGLS models were supplemented with analyses using ordinary least‐squares linear models. Following on the results of simple PGLS models, where significant predictors of genome size variation included six ecogeographical traits (see Table 1), we decided to fit a multiple PGLS model with a manual forward stepwise selection of ecogeographic variables to determine the optimal set of predictors (e.g., to see if other predictors can provide a significant amount of explained variation independent of that already provided by the first predictor included in the model). Only predictors that had a significant effect and resulted in the lowering of the model's Akaike information criterion (AIC) were included in the optimal PGLS model.

**TABLE 1:** Summary of statistical analyses aiming to explain variation in genome size among evolutionary lineages of the genus *Synura* using genomic, morphological, and environmental predictors. For comparative purposes, each statistical test was done separately using ordinary least‐squares models and models accounting for phylogenetic signal in the data. Significant values (*α* = 0.05) are reported in bold.

Explanatory variable	Ordinary linear models			Phylogeny‐corrected models			
	*F* statistic_(d.f.)_	*p*	Explained variation^a^ (%)	*F* statistic	*p*	Explained variation^a^ (%)	Lambda (*λ*)
Genomic GC content	0.65_(1,46)_	0.423	0.0	0.47 _(1,46)_	0.497	0.0	0.658
Cell size	20.24_(1,46)_	< **0.001**	29.1	17.84 _(1,46)_	< **0.001**	26.4	0.618
Scale size	0.90_(1,40)_	0.348	0.0	2.57 _(1,40)_	0.117	3.7	0.573
Scale length	6.85_(1,41)_	**0.012**	12.2	4.04 _(1,41)_	0.051	6.8	0.521
Presence of keel	23.0_(1,51)_	< **0.001**	29.7	3.24 _(1,51)_	0.078	4.1	0.545
Presence of hexagonal meshwork	0.05_(1,51)_	0.833	0.0	0.98 _(1,51)_	0.327	0.0	0.612
Presence of labyrinthine pattern	26.91_(1,51)_	< **0.001**	33.3	3.77 _(1,51)_	0.058	5.1	0.470
Water temperature	0.17_(1,46)_	0.682	0.0	0.73 _(1,46)_	0.399	0.0	0.624
Water pH	0.15_(1,47)_	0.698	0.0	0.00 _(1,47)_	0.973	0.0	0.618
Water conductivity	0.13_(1,45)_	0.723	0.0	0.09 _(1,45)_	0.771	0.0	0.595
Latitude	8.31_(1,51)_	**0.006**	12.3	8.36 _(1,51)_	**0.006**	12.4	0.606
Annual mean temperature (bio1)	5.41_(1,51)_	**0.024**	7.8	3.76 _(1,51)_	0.058	5.0	0.580
Mean diurnal range (bio2)	1.45_(1,51)_	0.235	0.8	0.00 _(1,51)_	0.955	0.0	0.651
Maximal temperature of warmest month (bio5)	0.20_(1,51)_	0.658	0.0	0.58 _(1,51)_	0.450	0.0	0.674
Mean temperature of wettest quarter (bio8)	6.27_(1,51)_	**0.015**	9.2	4.42 _(1,51)_	**0.040**	6.2	0.547
Mean temperature of driest quarter (bio9)	10.06_(1,51)_	**0.003**	14.8	6.58 _(1,51)_	**0.013**	9.7	0.521
Annual precipitation (bio12)	9.67_(1,51)_	**0.003**	14.3	4.23 _(1,51)_	**0.045**	5.9	0.531
Precipitation seasonality (bio15)	2.00_(1,51)_	0.164	1.9	1.35 _(1,51)_	0.252	0.7	0.626
Precipitation of warmest quarter (bio18)	2.63_(1,51)_	0.111	3.0	0.31 _(1,51)_	0.581	0.0	0.641
Bulk density (bdod)	0.07_(1,51)_	0.798	0.0	1.56 _(1,51)_	0.218	1.1	0.689
Cation exchange capacity (cec)	2.71_(1,51)_	0.106	3.2	3.88 _(1,51)_	0.054	5.2	0.605
Fraction of coarse fragments (cfvo)	4.73_(1,51)_	**0.034**	6.7	0.43 _(1,51)_	0.513	0.0	0.613
Proportion of clay particles (clay)	0.18_(1,51)_	0.671	0.0	0.04 _(1,51)_	0.838	0.0	0.655
Total nitrogen (nitrogen)	5.33_(1,51)_	**0.025**	7.7	9.00 _(1,51)_	**0.004**	13.3	0.685
Soil pH (phh2o)	1.12_(1,51)_	0.295	0.2	0.02 _(1,51)_	0.895	0.0	0.650
Proportion of sand particles (sand)	0.13_(1,51)_	0.718	0.0	0.64 _(1,51)_	0.428	0.0	0.669
Proportion of silt particles (silt)	1.17_(1,51)_	0.285	0.3	1.30 _(1,51)_	0.259	0.6	0.653
Soil organic carbon content (soc)	3.45_(1,51)_	0.069	4.5	5.34 _(1,51)_	**0.025**	7.7	0.671

^a^ Based on the adjusted *R* ^2^ coefficient.

## 3 Results

### 3.1 Genome Size and GC Content Diversity

**FIGURE 2:**
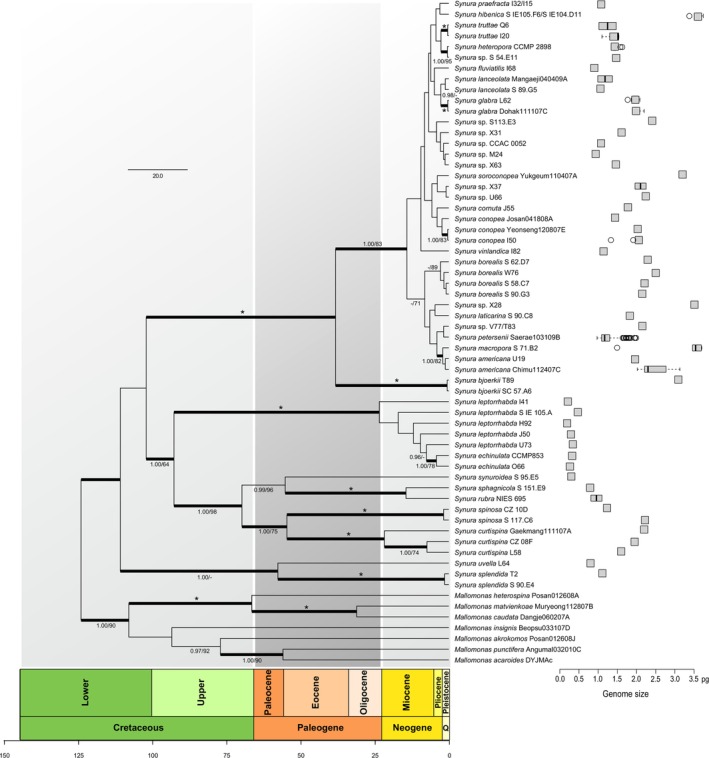
Time‐calibrated phylogenetic tree of the genus *Synura* based on nu ITS rDNA, nu SSU rDNA, and pt. *rbc*L sequence data with boxplots of genome size for each analyzed lineage. Values at the nodes indicate statistical support estimated by—MrBayes posterior node probability (left) and ML bootstrap (right). Only statistical supports higher than 0.95/60 are shown; 100/100 statistical support is marked by an asterisk. Scale bar—estimated number of substitutions per site. Genome size varies between strains from 0.19 to 3.70 pg.

This study was performed on 281 *Synura* strains obtained from 178 freshwater localities across the Northern hemisphere. Based on three molecular markers (nu ITS rDNA, nu SSU rDNA a pt. *rbc*L; Table S2), we identified 55 genotypes of the genus *Synura* and created a time‐calibrated phylogenetic tree (Figure 2). These ITS rDNA genotypes belong to 26 taxonomically accepted species and to 10 yet undescribed lineages. We successfully estimated genome size in all 98 newly obtained *Synura* strains (Table S4). The flow cytometric measurements were precise, resulting in clearly delimited peaks in FCM histograms, and the average coefficient of variation among repeated estimates was 1.0%. The genome size exhibited a 19‐fold difference between the *Synura* representatives with the lowest and the highest values, *S*. *leptorrhabda* H92 (0.19 pg/184 Mbp) and *S. macropora* 968 (3.70 pg/3.62 Gbp), respectively. Several *Synura* species analyzed in this study exhibited intraspecific genome size variability (Table S4). The most pronounced variability occurred within three species: *S. americana* (2.02–3.13 pg), *S*. *conopea* (1.32–2.11 pg) and *S. truttae* (1.02–1.54 pg).

The genomic DNA base composition (% GC content) differed by 15.3% between *Synura* strains with the lowest and the highest values, *S. borealis* W76 (34.0%) and *S. curtispina* CZ08F (49.3%), respectively.

### 3.2 Genome Size Evolution in the Genus Synura

**FIGURE 3:**
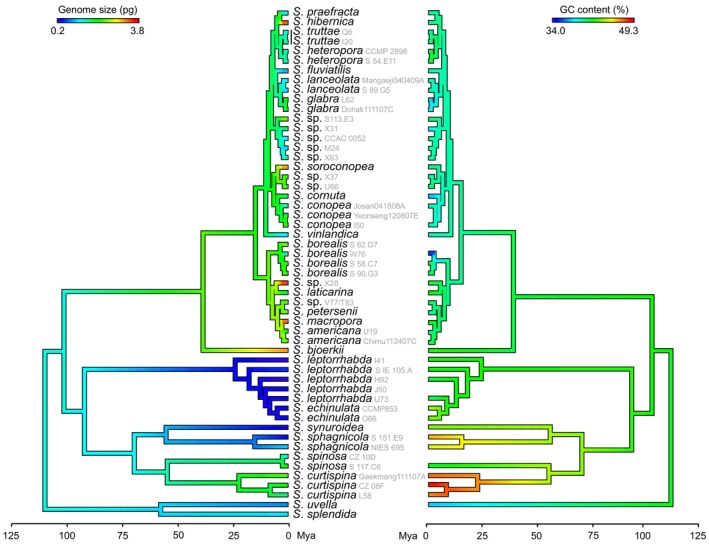
Evolution of genome size and GC content in the chrysophyte of the genus *Synura* as inferred using ancestral state reconstruction and mapped on a time‐calibrated phylogeny.

Reconstructed evolution of genome size in the genus *Synura* suggested that the ancestor had a rather lower genome size (1.27 pg) and a general tendency towards genome downsizing was apparent in most early diverging clades (Figure 3). Contrary to that, in the crown group, where most of the investigated taxa belong, genome downsizing or upsizing seemed to act in a lineage‐specific manner. In other words, trends of genome downsizing occasionally reach deeper into the phylogeny, whereas genome upsizing seems to be rather restricted to short terminal branches (e.g., divergence times < 10 Mya).

The patterns of genome size variation in *Synura* are not independent of phylogenetic relationships among the taxa, as evidenced by the measure of phylogenetic signal, *λ* = 0.65 (95% confidence limits = 0.29–0.89). Estimates of the parameter *λ* were significantly different from both 0 and 1 (*p* < 0.001 in each case). Therefore, phylogeny had a considerable effect on the distribution of genome size diversity within the genus, though other factors were also involved. A significant portion of the genome‐size variation could be explained by 11 out of 28 genomic, morphological, and ecogeographic predictors in independently conducted ordinary least‐squares models (Table 1). When phylogeny‐corrected PGLS models were applied, the number of significant predictors was reduced to seven and *λ* ranged 0.47–0.70 across the models (Table 1).

### 3.3 Morphological Correlates of Genome Size Variation

**FIGURE 4:**
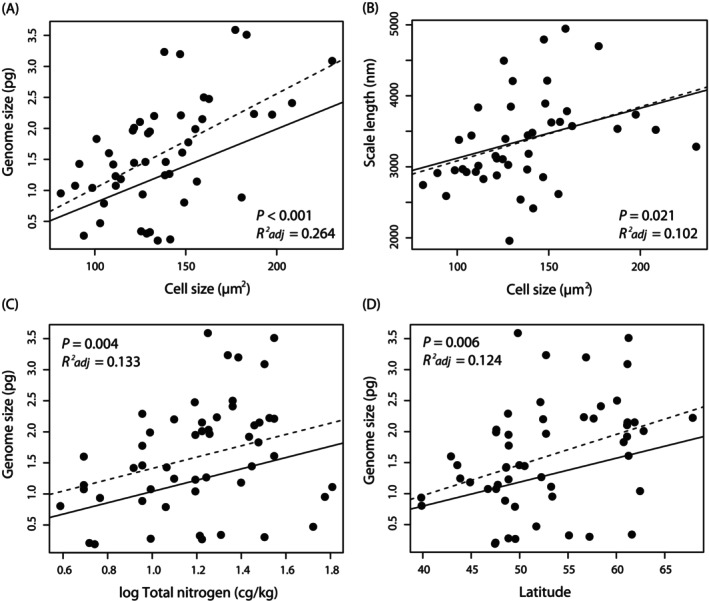
Variables directly or indirectly associated with genome size in the chrysophyte genus *Synura*. Regression lines are shown for both the ordinary least‐squares models (dashed line) and for the phylogeny‐corrected PGLS models (solid line), test statistics only for the latter.

Out of the four morphological predictors significantly associated with genome size variation in ordinary linear models (cell size, scale length, presence of keel, and presence of labyrinthine pattern), only cell size remained significant after the phylogenetic correction (Table 1). With 26.4% of explained variation (adjusted *R* ^2^ in the PGLS model), cell size was the most informative predictor of genome size among the 28 predictors tested in our study (Figure 4A).

Many morphological traits that were scored on siliceous scales exhibited phylogenetically conserved (i.e., clade specific) values, and their variation could be largely explained by the phylogeny (presence of keel, presence of labyrinthine pattern, presence of hexagonal meshwork). While scale size was not directly linked to the genome size variation (Table 1), scale length explained a significant amount of genome size variation in ordinary linear models (*F*_1,41_ = 6.85, *p* = 0.012, *R* ^2^_adj_ = 0.122) and its effect was marginally non‐significant after the phylogenetic correction (*F*_1,41_ = 4.04, *p* = 0.051, *R* ^2^_adj_ = 0.068). In phylogeny‐corrected models, both scale size and scale length were more closely associated with cell size (*F*_1,40_ = 4.68, *p* = 0.036, *R* ^2^_adj_ = 0.082; *F*_1,41_ = 5.77, *p* = 0.021, *R* ^2^_adj_ = 0.102, Figure 4B, respectively).

### 3.4 Ecogeographic Correlates of Genome Size Variation

To assess whether *Synura* lineages with small or large genomes have specific ecological requirements, each lineage was characterized by the values of 21 ecogeographic variables averaged across the sampling sites. Seven ecogeographic predictors were significantly associated with genome size variation in ordinary linear models (Table 1). Five of these remained significant when the phylogenetic correction was applied (latitude, mean temperature of wettest quarter, mean temperature of driest quarter, annual precipitation, total soil nitrogen) and one additional predictor became significant (fraction of coarse fragments; Table 1). Total soil nitrogen, latitude, and mean temperature of driest quarter were the most informative predictors in independently conducted PGLS analyses, explaining 13.3%, 12.4%, and 9.7% of the overall variation in genome size, respectively. In *Synura*, lineages possessing larger genome size seem to be associated with habitats with increased soil nitrogen content (Figure 4C), at higher latitudes (Figure 4D), and with lower mean temperatures in the driest quarter of the year.

When an AIC‐based forward stepwise selection of ecogeographic predictors was applied, total soil nitrogen was included in the PGLS model in the first step as the most informative predictor of genome size variation; however, no other predictor provided a significant amount of independent explained variation, not allowing for the fitting of a multiple PGLS model.

## 4 Discussion

The variation in genome size among eukaryotes and its phenotypic consequences are still largely unknown. This is particularly true for unicellular eukaryotes, for which genome size data are scarce. For the most well‐studied groups of organisms, plants and animals, genome size has been shown to be tightly linked with cell size, which may further relate to the ecology and evolution of species (e.g., Gregory 2002; Nardon et al. 2005; Leinaas et al. 2016; Pyšek et al. 2018; Trávníček et al. 2019; Simonin and Roddy 2018). In contrast to multicellular organisms, where the effects of genome size increase can be partially compensated by reducing the total number of cells, more pronounced phenotypic consequences might be expected in unicellular eukaryotes, where one cell represents the entire body. The variation in cell size resulting from genome size differences could, for example, be reflected in their efficiency of nutrient acquisition, light harvesting, selection of potential predators, and/or sedimentation rate (Finkel et al. 2001; Reynolds et al. 2002; Smetacek et al. 2004; Irwin et al. 2006), and thus fundamentally alter the ecological requirements of protist species.

In this study, we analyzed the genome size of 53 evolutionary lineages of the chrysophyte genus *Synura*. To account for the presumed phylogenetic dependence of traits among related species, we employed phylogeny‐corrected statistical models, which have rarely been applied to protists (e.g., Gray et al. 2007; Greenwold et al. 2019), possibly due to unknown evolutionary relationships among taxa. The number of genotypes included makes our study the most comprehensive attempt to explain genome size variation and its consequences in any protist group.

### 4.1 Genome Size and GC Content Vary Considerably

Species of the genus *Synura* show a significant variation in genome size, ranging from 0.19 to 3.70 pg among the taxa investigated in this study. Intermediate values of Pagel's coefficient *λ* in our analyses suggest that shared evolutionary histories may explain some, but not all, of the variation in genome size among *Synura* species. The reconstructed evolution of genome size in the genus *Synura* (Figure 3) revealed an interesting pattern: pronounced trends of genome size increases have occurred recently in several crown group taxa in the context of a much broader and older pattern of genome size reduction (also including other crown group representatives). From a macroevolutionary perspective, we can speculate that genome size increases may be less stable than genome size decreases, possibly due to ecological and evolutionary constraints associated with large genome (and cell) size. While this is consistent with previous studies showing that plant species with larger genomes are more prone to extinction (Vinogradov 2003; Soto Gomez et al. 2024), it is unclear to what extent this pattern can be applied to chrysophytes or protists in general. Another possible explanation for the relatively recent increase in genome size in some crown group lineages is that it coincides with the global decrease in temperature since the Middle Eocene (e.g., Zachos et al. 2001). Given that genome size is linked to cell size, this pattern would align with the “temperature‐size rule” (Montagnes and Franklin 2001; Atkinson et al. 2003), which predicts a 2.5% increase in protist cell size for every 1°C decrease in the mean temperature of the environments they inhabit. However, the temperature‐size rule alone cannot explain why increases and decreases in genome size were happening concurrently in different lineages of the crown group.

A genome‐size‐driven increase in cell size in *S*. *petersenii* was found to be associated with a reduced growth rate (Čertnerová and Škaloud 2020) and likely reduced nutrient uptake efficiency, both of which decrease their competitiveness. These factors increase the probability that strains with larger cells (and genomes) would be outcompeted by smaller cells and other microalgae, which could be a significant evolutionary constraint on large genome size in protists. Changes in genome size could be potentially quite dynamic, as suggested by the often contrasting trends in genome size evolution between sister lineages of *Synura* within the crown group as well as the incidence of intraspecific genome size variability detected within several taxa. Genome size differences maintained among populations of a species not only signal limited gene flow and possibly incipient speciation but also increase the opportunities for selection to act on genome size variation and its adaptive potential. Nonetheless, particular evolutionary mechanisms responsible for genome size variation in *Synura* remain unknown.

As with genome size, the GC content of *Synura* genomes varies widely (from 34.0% to 49.3%, Figure 3). Interestingly, the GC content variation observed here within a single microalgal genus is highly comparable to that of almost all monocot plants combined (i.e., 33.6%–48.9%, excluding orchids; Šmarda et al. 2014; Trávníček et al. 2019). Although genomic DNA base composition is predicted to significantly affect genome functioning and species ecology (Šmarda et al. 2014; Trávníček et al. 2019), it has only rarely been studied in protists, and the scarce GC content data are mostly derived from whole genome sequencing (e.g., Read et al. 2013; Majda et al. 2021). For example, Majda et al. (2021) analyzed GC content in 13 species of chrysophytes and found a range of 34.1%–54.5%, with higher GC content in heterotrophic taxa, which tend to have smaller genomes compared to their autotrophic counterparts (Olefeld et al. 2018; Majda et al. 2021). In our dataset, the higher GC content also tends to occur in taxa with smaller genomes, but genome size is not generally correlated with GC content. The species *S. curtispina* and *S. sphagnicola* have the highest genomic GC content (over 47%; this study; Čertnerová et al. 2022). Interestingly, the closest relative of *S. curtispina* , *S. spinosa* , has a much lower GC content (41.7%). Similarly, the lowest GC content was found in the *S. borealis* strain W76 (34.0%), while other *S. borealis* strains have much higher GC content (almost 41%). This suggests the potential for rapid changes in GC content during the evolution of *Synura* species.

### 4.2 Cell and Silica Scale Size Adjust to Changes in Genome Size

In addition to genomic variables, we analyzed 28 (of the initial pool of 39) morphological and ecogeographic variables, which were further tested as predictors of genome size in a phylogenetic context. Our results showed a strong association between genome size and cell size, explaining 29.1% and 26.4% of the variability in genome size in ordinary linear and phylogeny‐corrected models, respectively. However, the relationship was expected to be even stronger, as the cells are in direct contact with the environment and carry out all the functions. A likely explanation for this could be the relatively high variation in cell size measurements within strains (coefficients of variation reaching almost 20% on average). This is mainly due to the lack of a cell wall in chrysophytes, which allows high flexibility in changing cell size.

In addition to cell size being associated with genome size, we hypothesized that variation in these traits might also affect the size and ultrastructure (ornamentation) of the silica scales that cover the cell surface and form a compact envelope. Scale ornamentation appeared to be strongly phylogenetically conserved and independent of changes in genome (and cell) size. On the other hand, scale size and length were correlated with cell size, though these relationships were not very tight (Figure 4B; explaining 8% and 10% of the variability in cell size, respectively), possibly due to the high plasticity in cell size among the *Synura* strains. One of these variables, scale length, was even directly associated with genome size, although the relationship remained marginally non‐significant after applying phylogenetic correction. It appears that adjustments in silica scale length are more important in the context of changing cell size than those related to scale width or overall size (area). This is probably due to the fact that adjacent silica scales tend to overlap laterally to varying degrees when forming a compact cell envelope. The scale length could also play an important role in shaping the cells and fitting them into the colony. Overall, it seems that as genome size increases, the silica envelope must also adapt to accommodate the larger cell size.

### 4.3 Genome Size Is Linked to Ecological Preferences of Species

This study also sought to assess whether evolutionary changes in genome size led to shifts in the ecological preferences of protist species. To this end, we tested for associations between the genome size of 53 *Synura* lineages and averaged values of environmental variables that were either assessed directly at their original sites or extracted from publicly available databases. We took a conservative approach and only compiled data from sites where a flow cytometric genome size assessment had been performed on a strain that had been transferred to culture and taxonomically identified with a molecular barcode.

The best ecogeographic predictor of genome size variation in *Synura* was soil nitrogen content, which was positively associated with genome size and explained 13.3% of the variation in a phylogeny‐corrected PGLS model (Table 1). Since nucleic acids are among the most nitrogen (N) and phosphorus (P) demanding cellular biomolecules, it has been hypothesized that larger genomes are costly to build and maintain under nitrogen or phosphorus limitation (Hessen et al. 2010; Guignard et al. 2016). The “genome size‐nutrient interaction hypothesis”, which proposes that local availability of N and P can directly influence nucleic acid synthesis, is supported by evidence from plants. While polyploid plants with larger genomes dominate sites with higher N and P contents (Guignard et al. 2016), carnivorous plants, typically found in nutrient‐poor habitats, tend to have small genome sizes (Ellison 2006). In addition, increased N inputs increase the productivity primarily of grassland species with large genome sizes (Peng et al. 2022). Consistently, our results show that *Synura* lineages with larger genomes tend to occur in areas of higher soil nitrogen content. Interestingly, lineages with small genome sizes occur across the soil nitrogen content spectrum (Figure 4C), suggesting that the ecological constraint primarily affects their larger genome counterparts. It should be noted that database‐derived soil nitrogen content provides only an indirect indication of the actual availability of nitrogen in biologically relevant chemical forms dissolved in water, which has not been measured directly in situ. On the other hand, in areas with more nitrogen‐rich soil profiles, water flowing into reservoirs from the surrounding landscape can be expected to be enriched with nutrients.

The second most informative ecogeographic predictor was latitude, explaining altogether 12.4% of genome size variability in a PGLS model. *Synura* lineages with larger genomes were predominantly distributed at higher latitudes in the Northern Hemisphere (Figure 4D). This is consistent once again with the “temperature‐size rule” (see above), which states that protist cell size increases with a decrease in mean environmental temperatures. Latitude correlates with the most profound temperature gradient on the planet, and significant effects of two other temperature‐related variables in our PGLS models (Table 1) suggest the involvement of temperature differences among *Synura* inhabited sites in shaping the patterns of how genome size variation is distributed geographically. Furthermore, in colder areas (generally in higher latitudes), predators tend to have larger body sizes, which may be another factor selecting for increased genome and cell size in *Synura* lineages. A similar trend has already been observed, for example, in the geographical distribution of genome size in marine diatoms (Roberts et al. 2024).

While both soil nitrogen content and latitude (via temperature) can be well justified to determine the geographic distribution of *Synura* lineages with different genome sizes, our setup does not allow us to disentangle their relative importance. This is because the values of these variables are correlated in our dataset (*r* = 0.675, *p* < 0.001, Figure S1) and their explanatory potential largely overlaps; that is, if one predictor is already part of a PGLS model, including the other will not provide a significant amount of independent explained variation in an AIC‐based forward stepwise model selection. Competition experiments with *Synura* strains of different genome sizes cultivated under manipulated nutrient and/or temperature gradients are currently underway to test which of these ecological parameters is more likely to constrain the spatial distribution of genome size diversity in natural protist populations.

## 5 Conclusions

This study evaluated genome size variation in unicellular freshwater microalgae of the genus *Synura* and investigated its morphological and ecogeographic consequences in a phylogenetic framework. We show that the evolutionary increase in genome size is accompanied by an increase in cell size, as has been repeatedly shown in protists, but there is a noticeable dearth of studies exploring further evolutionary ramifications of this relationship. The narrower range of ecological conditions available to *Synura* lineages with larger genomes, together with signals of macroevolutionary instability of profound genome size increases, indicate that there may be an evolutionary cost to having a larger genome in protists. It remains unclear whether the accumulation of more nuclear DNA in *Synura* lineages (e.g., through the proliferation of selfish DNA elements) remains an effectively neutral mutation up to a certain threshold, determined by the biological limits of the species and/or the ecological context, or whether it might provide some adaptive benefits along the way. For example, coding DNA may be better shielded from mutations in larger genomes (Hsu 1975), larger cells (and genomes) in protists may serve as a pre‐adaptation to lower temperature habitats or allow them to escape the predator size spectrum (Lampe et al. 2021). On the other hand, *Synura* lineages with smaller genomes seemed to be more ecologically versatile, which could be the key to their presumably higher evolutionary success. It would be interesting to observe whether the lineages with smaller genomes (and faster dividing cells; Čertnerová and Škaloud 2020) will always outcompete their larger genome counterparts, or whether the lineages with larger genomes will perform better, at least under some specific environmental conditions (e.g., low temperature, excess nutrients). Our ongoing ex situ competition experiments with *Synura* strains of different genome sizes may shed light on ecological and microevolutionary processes operating in genome size‐variable planktonic communities of protists.

## Supporting information


Figure S1.

Table S1.

Table S2.

Table S3.

Table S4.

## Data Availability

The data that support the findings of this study are openly available in GenBank at https://www.ncbi.nlm.nih.gov/genbank/.
