# Supplementary material for: Large Genomes Are Associated With Greater Cell Size and Ecological Shift Towards More Nitrogen‐Rich and Higher‐Latitude Environments in Microalgae of the Genus Synura
Source: J Eukaryot Microbiol. 2025 Jul 2;72(4):e70026. doi: 10.1111/jeu.70026 (PMC12223332; doi:10.1111/jeu.70026)
Supplement: Supplementary file 1 — Figure S1. [file JEU-72-e70026-s004.docx]

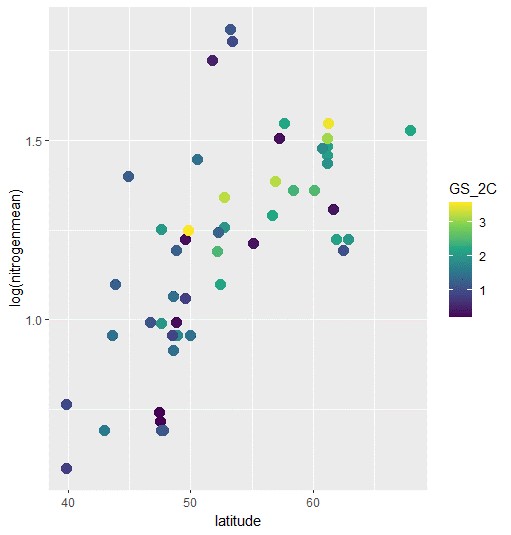


Figure S1 Correlation between the two most informative ecogeographic predictors of genome size variation in *Synura*, soil nitrogen content and latitude. The values were averaged across collection sites for each of the 53 evolutionary lineages of *Synura* included in this study.
