# Supplementary material for: Large Genomes Are Associated With Greater Cell Size and Ecological Shift Towards More Nitrogen‐Rich and Higher‐Latitude Environments in Microalgae of the Genus Synura
Source: J Eukaryot Microbiol. 2025 Jul 2;72(4):e70026. doi: 10.1111/jeu.70026 (PMC12223332; doi:10.1111/jeu.70026)
Supplement: Supplementary file 2 — Table S1. [file JEU-72-e70026-s003.docx]

Table S1. Collection details for newly obtained *Synura* strains for this study.

| Species | Strain | Collection site | GPS coordinates | Sampling date | *in situ* measured environmental variables | | |
| --- | --- | --- | --- | --- | --- | --- | --- |
|  |  |  |  |  | Water temperature (°C) | pH | Conductivity (µS cm^-1^) |
| *S. americana* | I52 | Unnamed pond, Flatrock, Newfoundland, Canada | 47°41'8.088"N, 52°43'14.772"W | 25.05.2017 | 8.0 | 7.3 | 213 |
|  | J40 | Unnamed pond, Newfoundland, Canada | 48°56'12.228"N, 55°30'16.848"W | 28.05.2017 | 11.0 | 8.1 | 112 |
|  | J7 | Lasso Pond, Newfoundland, Canada | 49°9'31.824"N, 55°3'57.060"W | 27.05.2017 | 11.0 | 7.7 | 75 |
|  | K2 | Expoits River oxbow lake, Newfoundland, Canada | 48°56'32.424"N, 55°46'9.336"W | 29.05.2017 | 11.0 | 7.8 | 37 |
|  | M30 | Saarineitämöjärvi pond, Inari, Finland | 68°37'49.469"N, 27°34'2.330"E | 02.10.2017 | 7.3 | 6.3 | 27 |
|  | M75 | Wiel van Collee, Leerdam, The Netherlands | 51°53'25.426"N, 5°6'14.011"E | 08.11.2017 | 9.9 | 7.6 | 621 |
|  | Q26 | Étang de Lacanau, Lacanau, France | 45°0'3.701"N, 1°8'14.201"W | 30.03.2018 | 13.0 | 7.5 | 230 |
|  | S39 | Raudtee Karjäär, Pärnumaa, Estonia | 58°30'31.824"N, 24°48'18.144"E | 20.04.2018 | 12.2 | 7.5 | 289 |
|  | S63.E10 | Turyňský rybník, Tuchlovice, Czech Republic | 50°8'47.292"N, 14°0'50.062"E | 19.03.2012 | 5.2 | 7.2 | 670 |
|  | U19 | Karer, Khanty-Mansiysky District, Russia | 61°6'25.956"N, 73°45'14.436"E | 28.05.2018 | 7.6 | 8.0 | 128 |
|  | V18 | Unnamed lake, Khanty-Mansiysky District, Russia | 61°8'28.860"N, 72°43'45.768"E | 27.06.2018 | 5.7 | 7.7 | 161 |
|  | X54 | Eramosa river, Guelph, Canada | 43°32'26.477"N, 80°14'22.027"W | 02.04.2018 | 5.1 | 8.4 | 700 |
| *S. bjoerkii* | T89 | Unnamed lake, Khanty-Mansiysky District, Russia | 61°8'44.736"N, 73°23'10.716"E | 28.05.2018 | 5.6 | 6.1 | 48 |
| *S. borealis* | J57 | Small Brook river, Newfoundland, Canada | 48°48'24.696"N, 55°31'54.804"W | 28.05.2017 | 13.0 | 7.5 | 32 |
|  | R85 | Emajõe-Suursoo wetland, Tartumaa, Estonia | 58°20'8.916"N, 27°15'49.788"E | 15.05.2018 | 7.6 | 6.6 | 36 |
|  | S58.C7 | Lillesjön lake, Växjö, Sweden | 56°57'19.548"N, 14°42'51.408"E | 22.04.2011 | 12.4 | 6.5 | 81 |
|  | S90.G3 | Harjujärvi, Joutsa, Finland | 61°53'7.688"N, 26°0'53.258"E | 05.05.2012 | 7.0 | 4.9 | - |
|  | W76 | Irtyš river, Khanty-Mansiysky District, Russia | 60°3'45.468"N, 69°54'27.612"E | 28.06.2018 | 13.3 | 8.7 | 328 |
| *S. conopea* | E71 | Beaver Lake, Washington, USA | 47°35'13.736"N, 122°0'6.257"W | 15.04.2017 | - | - | - |
|  | F27 | Trummen lake, Växjö, Sweden | 56°51'38.045"N, 14°49'26.551"E | 01.04.2017 | - | 6.8 | 298 |
|  | F35 | Vederslövssjön, Växjö, Sweden | 56°47'4.524"N, 14°44'15.911"E | 01.04.2017 | - | 6.9 | 112 |
|  | I29 | Quidi Vidi Lake, Newfoundland, Canada | 47°34'39.972"N, 52°41'48.300"W | 24.05.2017 | 9.0 | 7.4 | 775 |
|  | I50 | Long Pond, Newfoundland, Canada | 47°34'38.172"N, 52°44'1.392"W | 24.05.2017 | 9.0 | 7.4 | 690 |
|  | I57 | Shoe Cove Pond, Newfoundland, Canada | 47°44'30.696"N, 52°44'29.688"W | 25.05.2017 | 12.5 | 7.3 | 75 |
|  | I6 | First Pond, Newfoundland, Canada | 47°27'27.576"N, 52°43'46.272"W | 24.05.2017 | 8.0 | 7.4 | 77 |
|  | N70 | Lagoon of Río Támega, Mourazos, Spain | 41°53'12.552"N, 7°26'48.840"W | 06.02.2018 | 7.0 | 7.5 | 69 |
| *S. conopea* | N81 | Río Támega, Mourazos, Spain | 41°53'3.876"N, 7°26'42.900"W | 06.02.2018 | 7.2 | 7.1 | 62 |
|  | O17 | Albufeira da Barragem do Alto Cávado, Sezelhe, Portugal | 41°48'2.808"N, 7°52'33.996"W | 07.02.2018 | 4.1 | 7.2 | 29 |
|  | O32 | Albufeira da Venda Nova, Montalegre, Portugal | 41°40'38.316"N, 7°59'1.716"W | 07.02.2018 | 9.0 | 6.0 | 22 |
|  | S29.4 | Kladský rybník, Mariánské lázně, Czech Republic | 50°1'38.453"N, 12°40'28.862"E | NA | - | - | - |
|  | S7.10 | Velký Babín, Matějov, Czech Republic | 49°32'31.679"N, 15°53'48.638"E | NA | - | - | - |
|  | X46 | Pohořský rybník, Pohorská Ves, Czech Republic | 48°36'57.880"N, 14°40'34.427"E | 18.06.2018 | 19.0 | 6.5 | 37 |
| *S. cornuta* | J55 | Small Brook river, Newfoundland, Canada | 48°48'24.696"N, 55°31'54.804"W | 28.05.2017 | 13.0 | 7.5 | 32 |
|  | K15 | Unnamed lake, Newfoundland, Canada | 48°56'37.104"N, 55°49'23.844"W | 29.05.2017 | 10.0 | 7.4 | 39 |
| *S. curtispina* | CZ08F | Oxbow lake of Lužnice river, Horní Lužnice, Czech Republic | 48°51'9.324"N, 14°54'31.320"E | 12.03.2020 | 10.5 | 8.2 | 228 |
|  | L58 | Obština Botevgrad, Botevgrad, Bulgaria | 42°54'20.772"N, 23°48'55.584"E | 17.11.2017 | 8.6 | 8.0 | 387 |
|  | SAG29.92 | Nikolassee, Berlin, Germany | 52°25'25.684"N, 13°11'29.353"E | 1989 | - | - | - |
| *S. echinulata* | G65 | Unnamed pond, Larvik, Norway | 59°4'40.429"N, 10°2'23.388"E | 28.04.2017 | 7.0 | 7.4 | 274 |
|  | H47 | Damtjerna, Larvik, Norway | 59°4'51.618"N, 10°2'9.024"E | 28.04.2017 | 7.7 | 7.1 | 190 |
|  | L51 | Lacul Fânari, Fânari, Romania | 44°47'32.856"N, 26°5'6.036"E | 17.11.2017 | 8.2 | 8.4 | 720 |
| *S. echinulata* | O66 | Canal Limia, Porqueira, Spain | 42°1'15.528"N, 7°52'58.944"W | 07.02.2018 | 5.7 | 7.6 | 134 |
|  | U96 | Unnamed pond, Surgut - Lyantor, Khanty-Mansiysky District, Russia | 61°25'39.540"N, 72°47'7.368"E | 27.06.2018 | 3.6 | 6.2 | 76 |
|  | X16 | Unnamed pond next to Ödensee, Pichl-Kainisch, Austria | 47°33'48.896"N, 13°49'29.218"E | 14.06.2018 | 10.4 | 7.9 | 276 |
| *S. fluviatilis* | I68 | Shoe Cove Pond, Newfoundland, Canada | 47°44'30.696"N, 52°44'29.688"W | 25.05.2017 | 12.5 | 7.3 | 75 |
|  | J53 | Small Brook river, Newfoundland, Canada | 48°48'24.696"N, 55°31'54.804"W | 28.05.2017 | 13.0 | 7.5 | 32 |
|  | J87 | Expoits River oxbow lake, Newfoundland, Canada | 48°56'32.424"N, 55°46'9.336"W | 29.05.2017 | 11.0 | 7.8 | 37 |
| *S. hibernica* | 105.F6 | Caha Lakes, Curraduff, Ireland | 51°43'18.217"N, 9°39'37.001"W | 2010 | - | - | - |
|  | I54 | Gallows Cove Pond, Newfoundland, Canada | 47°40'47.712"N, 52°43'40.404"W | 25.05.2017 | 11.5 | 7.7 | 235 |
|  | I81 | Shoe Cove Pond, Newfoundland, Canada | 47°44'30.696"N, 52°44'29.688"W | 25.05.2017 | 12.5 | 7.3 | 75 |
|  | I89 | Paddys Pond, Newfoundland, Canada | 47°28'24.528"N, 52°52'45.084"W | 25.05.2017 | 8.0 | 7.6 | 117 |
|  | J84 | Unnamed pond, Newfoundland, Canada | 48°19'33.636"N, 55°28'8.796"W | 28.05.2017 | 18.0 | 7.6 | 18 |
|  | J88 | Expoits River oxbow lake, Newfoundland, Canada | 48°56'32.424"N, 55°46'9.336"W | 29.05.2017 | 11.0 | 7.8 | 37 |
|  | S103.D5 | Gowlaun Lough, Lehid, Ireland | 51°46'59.376"N, 9°45'54.108"W | 2010 | - | - | - |
|  | SIE104_D11 | Glanmore Lake, Kerry, Ireland | 51°44'3.520"N, 9°46'23.570"W | 2010 | - | - | - |
|  | X76 | Beaver Lake, Vancouver, Canada | 49°18'14.587"N, 123°8'20.087"W | 05.08.2018 | - | - | - |
| *S. lanceolata* | H88 | Pettee Pond, Massachusetts, USA | 42°11'10.572"N, 71°14'26.412"W | 21.05.2017 | 22.0 | 7.6 | 437 |
|  | S89.G5 | Unnamed pond next to Koululampi, Vehniä, Finland | 62°26'30.649"N, 25°40'58.026"E | 05.05.2012 | 10.0 | 6.5 | - |
| *S. laticarina* | R93 | Apna jõgi, Tartumaa, Estonia | 58°20'36.888"N, 27°16'3.900"E | 15.05.2018 | 8.7 | 7.3 | 104 |
|  | S90.C8 | Tehriselkä, Hirvensalmi, Finland | 61°45'23.490"N, 26°29'8.909"E | 05.05.2012 | 6.0 | 5.5 | - |
|  | T80 | Unnamed lake, Nemchinova, Khanty-Mansiysky District, Russia | 61°8'48.300"N, 73°38'36.600"E | 28.05.2018 | 13.5 | 7.5 | 98 |
|  | U17 | Karer, Khanty-Mansiysky District, Russia | 61°6'25.956"N, 73°45'14.436"E | 28.05.2018 | 7.6 | 8.0 | 128 |
|  | U93 | Unnamed pond, Surgut - Lyantor, Khanty-Mansiysky District, Russia | 61°25'39.540"N, 72°47'7.368"E | 30.05.2018 | 3.6 | 6.2 | 76 |
| *S. leptorrhabda* | H92 | First Pond, Newfoundland, Canada | 47°27'27.576"N, 52°43'46.272"W | 24.05.2017 | 8.0 | 7.4 | 77 |
|  | I13 | First Pond, Newfoundland, Canada | 47°27'27.576"N, 52°43'46.272"W | 24.05.2017 | 8.0 | 7.4 | 77 |
|  | I41 | Long Pond, Newfoundland, Canada | 47°34'38.172"N, 52°44'1.392"W | 24.05.2017 | 9.0 | 7.4 | 690 |
|  | J50 | Unnamed lake, Newfoundland, Canada | 48°50'37.032"N, 55°30'12.852"W | 28.05.2017 | 13.0 | 7.5 | 32 |
|  | SIE105A | Caha Lakes, Curraduff, Ireland | 51°43'18.217"N, 9°39'37.001"W | 2010 | - | - | - |
|  | U73 | Unnamed lake, Khanty-Mansiysky District, Russia | 61°35'58.776"N, 72°34'11.136"E | 30.05.2018 | 6.7 | 7.0 | 34 |
| *S. praefracta* | I32 | Quidi Vidi Lake, Newfoundland, Canada | 47°34'39.972"N, 52°41'48.300"W | 24.05.2017 | 9.0 | 7.4 | 775 |
| *S. rubra* | C74 | Lettererafroe Lough, Galway, Ireland | 53°22'28.452"N, 9°25'0.552"W | 04.02.2017 | 6.0 | 6.3 | 78 |
|  | NIES 695 | Miyatoko Mire Fukushima, Japan | NA | NA | - | - | - |
| *S. sp.* | E22 | Andrew's Mare Lake, Lyndhurst, UK | 50°53'55.500"N, 1°38'29.868"W | 04.02.2017 | 11.0 | 6.8 | 45 |
|  | L65 | Unnamed pond near Parakalamos, Kalpaki, Greece | 39°52'28.596"N, 20°34'59.052"E | 18.11.2017 | 13.7 | 7.2 | 298 |
|  | M24 | Unnamed pond in Pindos, Metsovo, Greece | 39°47'58.524"N, 21°9'36.684"E | 20.11.2017 | 6.5 | 7.6 | 178 |
|  | S113.E3 | Emajõgi river, Tartumaa, Estonia | 58°23'46.903"N, 26°18'39.395"E | 10.05.2013 | 16.0 | 7.5 | 322 |
|  | S54.E11 | Klejnarka, Nové dvory, Czech Republic | 49°58'10.574"N, 15°19'16.345"E | 19.04.2011 | - | - | - |
|  | T35 | Pyreneje, 4, Canton des Pyrénées catalanes, France | 42°32'40.128"N, 2°5'25.224"E | 08.06.2018 | 14.4 | 7.4 | 44 |
|  | T83 | Oxbow lake of river Ob, Surgutsky District, Russia | 61°8'48.300"N, 73°38'36.600"E | 28.05.2018 | 13.5 | 7.5 | 98 |
|  | U20 | Karer, Khanty-Mansiysky District, Russia | 61°6'25.956"N, 73°45'14.436"E | 28.05.2018 | 7.6 | 8.0 | 128 |
|  | U66 | Oxbow lake of river Ob, Rechnik, Khanty-Mansiysky District, Russia | 61°15'39.528"N, 73°33'51.516"E | 29.05.2018 | 7.8 | 7.4 | 137 |
|  | V17 | Quidi Vidi Lake, Newfoundland, Canada | 47°34'39.972"N, 52°41'48.300"W | 31.05.2018 | 5.7 | 7.7 | 161 |
|  | V41 | Unnamed lake, Khanty-Mansiysky District, Russia | 60°57'15.840"N, 68°39'30.420"E | 27.06.2018 | 11.3 | 7.9 | 265 |
|  | X28 | Oxbow lake of river Ob, Rechnik, Khanty-Mansiysky District, Russia | 61°15'39.528"N, 73°33'51.516"E | 29.05.2018 | 7.8 | 7.4 | 137 |
|  | X31 | Oxbow lake of river Ob, Rechnik, Khanty-Mansiysky District, Russia | 61°15'39.528"N, 73°33'51.516"E | 29.05.2018 | 7.8 | 7.4 | 137 |
|  | X37 | Flooded valley in Khanty-Mansiysky District, Russia | 61°15'40.5"N, 73°37'07.8"E | 11.07.2018 | 9.4 | 7.1 | 59 |
|  | X63 | Clythe Creek, Guelph, Ontario, Canada | 43°33'31.932"N, 80°12'50.486"W | 27.04.2018 | 10.0 | 8.1 | 701 |
| *S. spinosa* | CZ10D | Lužnice river, Horní Lužnice, Czech Republic | 48°51'12.060"N, 14°54'22.608"E | 13.03.2020 | 8.4 | 7.6 | 220 |
|  | S117.C6 | Vidnarjaure, Kiruna, Sweden | 67°53'1.194"N, 18°59'57.041"E | 17.06.2013 | 12.0 | 6.6 | 19 |
| *S. splendida* | T2 | Unnamed channel, Gmina Trzcianne, Poland | 53°15'36.324"N, 22°36'12.852"E | 16.04.2018 | 16.9 | 7.0 | 190 |
| *S. synuroidea* | S95E5 | Unmaned lake, Nethy Bridge, Scotland, UK | 57°13'32.549"N, 3°43'20.708"W | 19.09.2012 | 11.0 | 5.9 | 27 |
| *S. truttae* | E29 | Unnamed pond, Loqueffret, France | 48°20'40.380"N, 3°51'37.728"W | 15.03.2017 | 11.0 | 7.0 | 63 |
|  | I20 | Quidi Vidi Lake, Newfoundland, Canada | 47°34'39.972"N, 52°41'48.300"W | 24.05.2017 | 9.0 | 7.4 | 775 |
|  | I30 | Quidi Vidi Lake, Newfoundland, Canada | 47°34'39.972"N, 52°41'48.300"W | 24.05.2017 | 9.0 | 7.4 | 775 |
|  | Q6 | Lac de Layres, Hourtin, France | 45°12'47.002"N, 1°6'55.238"W | 30.03.2018 | 13.0 | 7.4 | 236 |
|  | S34.1 | Unnamed pond, Mezní Louka, Czech Republic | 50°52'37.996"N, 14°19'10.614"E | NA | - | - | - |
|  | T61 | Étang du Ticou, Bolquère, France | 42°30'42.120"N, 2°4'12.360"E | 08.06.2018 | 10.0 | 8.2 | 110 |
| *S. uvella* | L64 | Unnamed pond near Parakalamos, Kalpaki, Greece | 39°52'28.596"N, 20°34'59.052"E | 18.11.2017 | 13.7 | 7.2 | 298 |
| *S. vinlandica* | I82 | Shoe Cove Pond, Newfoundland, Canada | 47°44'30.696"N, 52°44'29.688"W | 25.05.2017 | 12.5 | 7.3 | 75 |
