# Supplementary material for: Large Genomes Are Associated With Greater Cell Size and Ecological Shift Towards More Nitrogen‐Rich and Higher‐Latitude Environments in Microalgae of the Genus Synura
Source: J Eukaryot Microbiol. 2025 Jul 2;72(4):e70026. doi: 10.1111/jeu.70026 (PMC12223332; doi:10.1111/jeu.70026)
Supplement: Supplementary file 3 — Table S2. [file JEU-72-e70026-s001.docx]

Table S2. GenBank accession numbers for nuclear ITS rDNA, SSU rDNA and pt *rbc*L sequences of investigated *Synura* strains and *Mallomonas* strains used as an outgroup. New sequences are indicated in bold type.

| Species | Strain | GenBank accession number | | |
| --- | --- | --- | --- | --- |
|  |  | nu ITS rDNA | nu SSU rDNA | pt *rbc*L |
| *S. americana* | Chimu112407C | KP268712 | KM590551 | KM590838 |
|  | I52 | MN782177 | - | - |
|  | J40 | **PQ047261** | - | - |
|  | J7 | **PQ047262** | - | - |
|  | K2 | **PQ047263** | - | - |
|  | M30 | **PQ047264** | - | - |
|  | M75 | **PQ047265** | - | - |
|  | Q26 | **PQ047266** | - | - |
|  | S39 | **PQ047267** | - | - |
|  | S63.E10 | **PQ047268** | - | - |
|  | U19 | **PQ047269** | **PQ039822** | **PQ048856** |
|  | V18 | **PQ047270** | - | - |
|  | X54 | **PQ047271** | - | - |
| *S. bjoerkii* | T89 | **PQ047272** | **PQ039823** | **PQ048857** |
| *S. borealis* | J57 | MN782182 | - | - |
|  | R85 | **PQ047273** | - | - |
|  | S58.C7 | HG514168 |  | HG514234 |
|  | S62D7 | HG514176 | - | HG514235 |
|  | S90.G3 | **PQ047274** | - | **PQ048858** |
|  | W76 | **PQ047275** | - | **PQ048859** |
| *S. conopea* | E71 | **PQ047276** |  |  |
|  | F27 | **PQ047277** |  |  |
|  | F35 | **PQ047278** |  |  |
|  | I29 | MN782186 |  |  |
|  | I50 | **PQ047279** |  | MN783111 |
|  | I57 | **PQ047280** |  |  |
|  | I6 | **PQ047281** |  |  |
|  | Josan041808A | KP268691 | KM590556 | KM590843 |
|  | N70 | **PQ047282** |  |  |
|  | N81 | **PQ047283** |  |  |
|  | O17 | **PQ047284** |  |  |
|  | O32 | **PQ047285** |  |  |
|  | S29.4 | **PQ047286** |  |  |
|  | S7.10 | FM178506 |  |  |
|  | X46 | **PQ047287** |  |  |
|  | Yeonseong120807E | KP268689 | KM590558 | KM590845 |
| *S. cornuta* | J55 | MN782211 | **PQ039824** | MN783123 |
|  | K15 | **PQ047288** |  |  |
| *S. curtispina* | CZ08F | **PQ047289** | - | **PQ048860** |
|  | Gaekmang111107A | KP268734 | KM590559 | KM590846 |
|  | L58 | **PQ047290** | **PQ039825** | **PQ048861** |
|  | SAG29.92 | GU338151 |  |  |
| *S. echinulata* | CCMP853 | KP268754 | KM590563 | KM590850 |
|  | G65 | **PQ047291** |  |  |
|  | H47 | **PQ047292** |  |  |
|  | L51 | **PQ047293** |  |  |
|  | O66 | **PQ047294** | **PQ039826** | **PQ048862** |
|  | U96 | **PQ047295** |  |  |
|  | X16 | **PQ047296** |  |  |
| *S. fluviatilis* | I68 | MN782207 | **PQ039827** | MN783120 |
|  | J53 | MN782208 |  |  |
|  | J87 | MN782209 |  |  |
| *S. glabra* | C46 | OL803933 |  |  |
|  | C54 | OL803934 |  |  |
|  | Dohak111107C | KP268721 | JX455149 | JX455145 |
|  | E98 | OL803935 |  |  |
|  | F20 | OL803936 |  |  |
|  | F41 | OL803937 |  |  |
|  | F81 | OL803939 |  |  |
|  | G12 | OL803941 |  |  |
|  | G14 | OL803942 |  |  |
|  | G46 | OL803943 |  |  |
|  | G56 | OL803945 |  |  |
|  | G72 | OL803946 |  |  |
|  | G98 | OL803947 |  |  |
|  | H17 | OL803948 |  |  |
|  | K67 | OL803950 |  |  |
|  | K76 | OL803952 |  |  |
|  | L26 | OL803955 |  |  |
|  | L62 | OL803956 | **PQ039828** | **PQ048863** |
|  | L70 | OL803957 |  |  |
|  | L96 | OL803958 |  |  |
|  | M73 | OL803959 |  |  |
|  | M88 | OL803960 |  |  |
|  | O60 | OL803961 |  |  |
| *S. heteropora* | C11 | OL803964 |  |  |
|  | C38 | OL803965 |  |  |
|  | C71 | OL803966 |  |  |
|  | CCMP 2898 | GU338136 | GU325596 | GU325498 |
|  | D12 | OL803968 |  |  |
|  | D24 | OL803969 |  |  |
|  | F14 | OL803970 |  |  |
|  | F65 | OL803971 |  |  |
|  | F83 | OL803972 |  |  |
|  | K28 | OL803973 |  |  |
|  | L66 | OL803974 |  |  |
|  | N30 | OL803975 |  |  |
|  | S20.45 / CAUP B 709 | HG514206 |  |  |
| *S. hibernica* | 105.F6 | HG514219 |  |  |
|  | I54 | **PQ047297** |  |  |
|  | I81 | **PQ047298** |  |  |
|  | I89 | **PQ047299** |  |  |
|  | IE.B11 = Irsko IE-B11 | HG514206 |  |  |
|  | J84 | MN782189 |  |  |
|  | J88 | **PQ047300** |  |  |
|  | S103.D5 | **PQ047301** |  |  |
|  | SIE104_D11 | HG514216 | **PQ039829** | HG514248 |
|  | X76 | **PQ047302** |  |  |
| *S. lanceolata* | H88 | **PQ047303** |  |  |
|  | I37 | OL803978 |  |  |
|  | Mangaeji040409A | KP268700 | KM590577 | KM590864 |
|  | S89.G5 | **PQ047304** | **PQ039830** | **PQ048864** |
| *S. laticarina* | R93 | **PQ047305** |  |  |
|  | S90.C8 | HG514222 | **PQ039831** | HG514250 |
|  | T80 | **PQ047306** |  |  |
|  | U17 | **PQ047307** |  |  |
|  | U93 | **PQ047308** |  |  |
| *S. leptorrhabda* | H92 | MN782220 | MN782173 | MN783129 |
|  | I13 | MN782222 |  |  |
|  | I41 | MN782223 | MN782174 | MN783130 |
|  | J50 | MN782226 | MN782175 | MN783131 |
|  | SIE105A | KP268753 | HF549065 | HF549076 |
|  | U73 | **PQ047309** | **PQ039832** | **PQ048865** |
| *S. macropora* | 968 | OL803979 |  |  |
|  | M39 | OL803980 |  |  |
|  | S71.B4 | OL803981 |  |  |
|  | S71B2 |  | KM590582 | KM590869 |
|  | T66 | HG514228 |  |  |
|  | V29 | OL803983 |  |  |
|  | X40 | OL803984 |  |  |
| *S. petersenii* | Saeraeul103109B | KP268704 | KM590588 | KM590875 |
| *S. praefracta* | I32 | **PQ047310** |  |  |
| *S. rubra* | C74 | **PQ047311** |  |  |
|  | NIES 695 | OP737840 | OP700307 | OP719269 |
| *S. soroconopea* | F31 | OL803986 |  |  |
|  | Yukgeum110407A |  |  |  |
| *S. sp.* | CCAC0052 | GU338146 | GU325606 | GU325508 |
|  | E22 | **PQ047312** |  |  |
|  | L65 | **PQ047313** |  |  |
|  | M24 | **PQ047314** | **PQ039833** | **PQ048866** |
|  | S113.E3 | **PQ047315** | **PQ039834** | **PQ048867** |
|  | S54.E11 | HG514200 | **PQ039835** | **PQ048868** |
|  | T35 | **PQ047316** |  |  |
|  | T83 | OL803930 | - | - |
|  | U20 | **PQ047317** |  |  |
|  | U66 | **PQ047318** | **PQ039836** | **PQ048869** |
|  | V17 | **PQ047319** |  |  |
|  | V41 | **PQ047320** | - | - |
|  | X28 | **PQ047321** | **PQ039837** | **PQ048870** |
|  | X31 | **PQ047322** | **PQ039838** | **PQ048871** |
|  | X37 | **PQ047323** | **PQ039839** | **PQ048872** |
|  | X63 | **PQ047324** | **PQ039840** | **PQ048873** |
| *S. sphagnicola* | H73 | OL803987 |  |  |
|  | J54 / S150.D10 | OL803988 |  |  |
|  | K33 | MK322795 |  |  |
|  | K35 | OL803991 |  |  |
|  | K40 | OL803992 |  |  |
|  | K46 | MK322785 |  |  |
|  | K8 / S152.E5 | MK322792 |  |  |
|  | L0234 | MK322785 |  |  |
|  | M44 | MN782218 |  |  |
|  | S151E9_J82 | MK322794 | MK322771 | MK322930 |
| *S. spinosa* | CZ10D | **PQ047325** | **PQ039841** | **PQ048874** |
|  | S117.C6 | **PQ047326** |  | **PQ048875** |
| *S. splendida* | S90E4 | KP268761 | HF549071 | HF549082 |
|  | T2 | **PQ047327** |  |  |
| *S. synuroidea* | S95E5 | MN782219 | KX815882 | KX815884 |
| *S. truttae* | E29 | **PQ047328** |  |  |
|  | I20 | MN782203 | **PQ039842** | MN783117 |
|  | I30 | **PQ047329** |  |  |
|  | Q6 | **PQ047330** | **PQ039843** | **PQ048876** |
|  | S34.1 | **PQ047331** |  |  |
|  | T61 | **PQ047332** |  |  |
| *S. uvella* | L64 | **PQ047333** | **PQ039844** | **PQ048877** |
| *S. vinlandica* | I82 | MN782206 | **PQ039845** | MN783119 |
| *M. acaroides* | DYJMAc |  | JX946333 | JX946349 |
| *M. akrokomos* | Posan012608J |  | GU935625 | GU935667 |
| *M. caudata* | Dangje060207A |  | GU935629 | GU935671 |
| *M. heterospina* | Posan012608A |  | GU935617 | GU935659 |
| *M. insignis* | Beopsu033107D |  | GU935634 | GU935676 |
| *M. matvienkoae* | Muryeong112807B |  | GU935628 | GU935670 |
| *M. punctifera* | Angumal032010C |  | JQ955667 | JQ955662 |
