# Supplementary material for: Large Genomes Are Associated With Greater Cell Size and Ecological Shift Towards More Nitrogen‐Rich and Higher‐Latitude Environments in Microalgae of the Genus Synura
Source: J Eukaryot Microbiol. 2025 Jul 2;72(4):e70026. doi: 10.1111/jeu.70026 (PMC12223332; doi:10.1111/jeu.70026)
Supplement: Supplementary file 4 — Table S3. [file JEU-72-e70026-s002.docx]

Table S3. Values of six morphological traits analysed in *Synura* strains used in this study measured on 30 cells / scales.

| Species | Strain | Cell size  (µm^2^) | Scale size  (µm^2^) | Scale length  (µm) | Presence of keel | Presence of hexagonal meshwork | Presence of labyrinthine pattern |
| --- | --- | --- | --- | --- | --- | --- | --- |
| *S. americana* | M75 | 162.75 ± 30.7 | 6.24 | 3.57 | 1 | 0 | 0 |
| *S. americana* | U19 | 129.26 ± 13.9 | 6.68 | 3.85 | 1 | 0 | 0 |
| *S. bjoerkii* | T89 | 230.50 ± 57.9 | 5.28 | 3.28 | 1 | 0 | 0 |
| *S. borealis* | S58.C7 | 147.35 ± 23.0 | 9.19 | 4.79 | 1 | 0 | 0 |
| *S. borealis* | S90.G3 | 159.12 ± 29.7 | 9.42 | 4.94 | 1 | 0 | 0 |
| *S. borealis* | W76 | 159.92 ± 22.5 | 6.37 | 3.78 | 1 | 0 | 0 |
| *S. conopea* | I50 | 155.23 ± 20.2 | 3.12 | 2.62 | 1 | 0 | 0 |
| *S. conopea* | X46 | 91.51 ± 13.8 | - | - | 1 | 0 | 0 |
| *S. cornuta* | K15 | 151.36 ± 28.5 | 5.02 | 3.62 | 1 | 0 | 0 |
| *S. curtispina* | CZ08F | 130.31 ± 26.9 | 9.64 | 4.21 | 0 | 1 | 0 |
| *S. curtispina* | L58 | 107.74 ± 23.9 | 6.71 | 3.44 | 0 | 1 | 0 |
| *S. curtispina* | SAG29.92 | 132.68 ± 30.2 | - | - | 0 | 1 | 0 |
| *S. echinulata* | L51 | 130.30 ± 22.5 | - | - | 0 | 0 | 1 |
| *S. echinulata* | O66 | 93.97 ± 25.9 | 4.30 | 2.59 | 0 | 0 | 1 |
| *S. fluviatilis* | I68 | 180.72 ± 46.6 | - | - | 1 | 0 | 0 |
| *S. glabra* | L62 | 120.92 ± 18.1 | 5.18 | 3.15 | 1 | 0 | 0 |
| *S. glabra* | K67 | 121.58 ± 20.6 | 4.14 | 2.88 | 1 | 0 | 0 |
| *S. heteropora* | K28 | 123.88 ± 20.6 | 3.96 | 3.12 | 1 | 0 | 0 |
| *S. hibernica* | 105.F6 | 177.26 ± 40.9 | 8.44 | 4.70 | 1 | 0 | 0 |
| *S. lanceolata* | H88 | 114.31 ± 20.2 | 3.05 | 2.83 | 1 | 0 | 0 |
| *S. lanceolata* | S89.G5 | 98.64 ± 20.5 | 3.52 | 2.95 | 1 | 0 | 0 |
| *S. laticarina* | S90.C8 | 100.96 ± 30.1 | 5.76 | 3.38 | 1 | 0 | 0 |
| *S. leptorrhabda* | H92 | 134.59 ± 45.5 | 3.95 | 2.54 | 0 | 0 | 1 |
| *S. leptorrhabda* | I41 | 141.48 ± 33.1 | 4.05 | 2.42 | 0 | 0 | 1 |
| *S. leptorrhabda* | J50 | - | - | - | 0 | 0 | 1 |
| *S. leptorrhabda* | SIE105A | 102.97 ± 18.5 | 5.04 | 2.97 | 0 | 0 | 1 |
| *S. leptorrhabda* | U73 | 125.43 ± 28.7 | 12.47 | 4.49 | 0 | 0 | 1 |
| *S. macropora* | S71.B2 | 138.19 ± 24.9 | 4.40 | 2.96 | 1 | 0 | 0 |
| *S. petersenii* | B24 | 141.00 ± 21.8 | 5.38 | 3.48 | 1 | 0 | 0 |
| *S. praefracta* | I32 | 89.18 ± 14.2 | 3.65 | 2.91 | 1 | 0 | 0 |
| *S. rubra* | NIES 695 | 81.31 ± 13.0 | 5.02 | 2.74 | 0 | 0 | 0 |
| *S. soroconopea* | F31 | 146.90 ± 40.6 | 2.99 | 2.85 | 1 | 0 | 0 |
| *S. sp.* | M24 | 126.34 ± 25.5 | 5.02 | 3.39 | 1 | 0 | 0 |
| *S. sp.* | S113.E3 | 208.47 ± 33.1 | 6.11 | 3.52 | 1 | 0 | 0 |
| *S. sp.* | S54.E11 | 138.95 ± 23.1 | 3.86 | 3.18 | 1 | 0 | 0 |
| *S. sp.* | T35 | 111.50 ± 15.1 | 3.87 | 3.02 | 1 | 0 | 0 |
| *S. sp.* | T83 | 124.81 ± 17.8 | 4.06 | 3.11 | 1 | 0 | 0 |
| *S. sp.* | U66 | 187.52 ± 42.3 | 4.88 | 3.54 | 1 | 0 | 0 |
| *S. sp.* | X28 | 183.48 ± 37.2 | - | - | 1 | 0 | 0 |
| *S. sp.* | X31 | 148.05 ± 31.5 | 6.02 | 3.89 | 1 | 0 | 0 |
| *S. sp.* | X37 | 101.59 ± 22.9 | 5.55 | 3.59 | 1 | 0 | 0 |
| *S. sp.* | X63 | 127.81 ± 17.2 | 4.46 | 3.03 | 1 | 0 | 0 |
| *S. sphagnicola* | K35 | 105.05 ± 20.6 | 5.76 | 2.93 | 0 | 0 | 0 |
| *S. spinosa* | CZ10D | 111.43 ± 18.4 | 8.63 | 3.84 | 0 | 1 | 0 |
| *S. spinosa* | S117.C6 | 197.53 ± 37.2 | 6.23 | 3.73 | 0 | 1 | 0 |
| *S. splendida* | T2 | - | - | - | 0 | 0 | 0 |
| *S. synuroidea* | S95E5 | 128.50 ± 32.8 | 1.58 | 1.96 | 0 | 0 | 0 |
| *S. truttae* | I20 | 110.12 ± 22.6 | 4.00 | 2.93 | 1 | 0 | 0 |
| *S. truttae* | Q6 | 138.52 ± 34.1 | 5.43 | 3.44 | 1 | 0 | 0 |
| *S. uvella* | L64 | 149.15 ± 24.4 | 12.98 | 4.21 | 0 | 1 | 0 |
| *S. vinlandica* | I82 | 156.13 ± 36.0 | 5.02 | 3.63 | 1 | 0 | 0 |
