# Supplementary material for: Large Genomes Are Associated With Greater Cell Size and Ecological Shift Towards More Nitrogen‐Rich and Higher‐Latitude Environments in Microalgae of the Genus Synura
Source: J Eukaryot Microbiol. 2025 Jul 2;72(4):e70026. doi: 10.1111/jeu.70026 (PMC12223332; doi:10.1111/jeu.70026)
Supplement: Supplementary file 5 — Table S4. [file JEU-72-e70026-s005.docx]

Table S4. Nuclear DNA contents (reported in absolute units per cell) and genomic GC composition (%) of investigated *Synura* strains including standard deviation (SD) and the plant reference standards.

| Species | Strain | DNA content (pg) | GC content (%) | Reference standard |
| --- | --- | --- | --- | --- |
| *S. americana* | I52 | 2.33 ± 0.02 |  | *Bellis perennis* |
|  | J40 | 2.30 ± 0.05 |  | *Bellis perennis* |
|  | J7 | 2.30 ± 0.02 |  | *Bellis perennis* |
|  | K2 | 2.26 ± 0.03 |  | *Bellis perennis* |
|  | M30 | 2.16 ± 0.02 |  | *Bellis perennis* |
|  | M75 | 2.51 ± 0.03 | 46.1 ± 0.21 | *Bellis perennis* |
|  | Q26 | 2.02 ± 0.03 | 41.3 ± 0.20 | *Solanum pseudocapsicum* |
|  | S39 | 3.10 ± 0.05 | 41.3 ± 0.17 | *Solanum pseudocapsicum* |
|  | S63.E10 | 3.03 ± 0.02 | 41.5 ± 0.06 | *Solanum pseudocapsicum* |
|  | U19 | 1.92 ± 0.01 | 39.7 ± 0.14 | *Solanum pseudocapsicum* |
|  | V18 | 3.13 ± 0.04 |  | *Solanum pseudocapsicum* |
|  | X54 | 2.10 ± 0.01 |  | *Solanum pseudocapsicum* |
| *S. bjoerkii* | T89 | 3.09 ± 0.01 | 42.4 ± 0.71 | *Pisum sativum* cv. Ctirad |
| *S. borealis* | J57 | 2.29 ± 0.03 |  | *Bellis perennis* |
|  | R85 | 2.19 ± 0.02 | 41.4 ± 0.25 | *Bellis perennis* |
|  | S58.C7 | 2.23 ± 0.02 | 41.6 ± 0.52 | *Bellis perennis* |
|  | S90.G3 | 2.15 ± 0.03 | 40.9 ± 0.15 | *Bellis perennis* |
|  | W76 | 1.25 ± 0.02 | 34.0 ± 0.37 | *Bellis perennis* |
| *S. conopea* | E71 | 2.03 ± 0.01 |  | *Solanum pseudocapsicum* |
|  | F27 | 2.07 ± 0.03 | 38.8 ± 0.11 | *Solanum pseudocapsicum* |
|  | F35 | 2.05 ± 0.01 |  | *Solanum pseudocapsicum* |
|  | I29 | 2.11 ± 0.02 |  | *Solanum pseudocapsicum* |
|  | I50 | 2.11 ± 0.03 |  | *Solanum pseudocapsicum* |
|  | I57 | 2.05 ± 0.01 | 43.9 ± 0.14 | *Bellis perennis* |
|  | I6 | 1.90 ± 0.03 |  | *Solanum pseudocapsicum* |
|  | N70 | 2.02 ± 0.03 | 39.1 ± 0.17 | *Solanum pseudocapsicum* |
|  | N81 | 2.05 ± 0.03 |  | *Solanum pseudocapsicum* |
|  | O17 | 2.07 ± 0.02 | 38.9 ± 0.24 | *Solanum pseudocapsicum* |
|  | O32 | 2.01 ± 0.03 |  | *Solanum pseudocapsicum* |
|  | S29.4 | 1.32 ± 0.01 | 43.5 ± 0.19 | *Bellis perennis* |
|  | S7.10 | 2.11 ± 0.02 |  | *Solanum pseudocapsicum* |
|  | X46 | 1.43 ± 0.02 | 39.5 ± 0.10 | *Solanum pseudocapsicum* |
| *S. cornuta* | J55 | 1.76 ± 0.02 | 37.9 ± 0.17 | *Solanum pseudocapsicum* |
|  | K15 | 1.79 ± 0.03 | 36.9 ± 0.14 | *Solanum pseudocapsicum* |
| *S. curtispina* | CZ08F | 1.95 ± 0.02 | 49.3 ± 0.12 | *Carex acutiformis* |
|  | L58 | 1.60 ± 0.01 | 47.5 ± 0.28 | *Bellis perennis* |
|  | SAG29.92 | 2.20 ± 0.02 | 47.8 ± 0.32 | *Bellis perennis* |
| *S. echinulata* | G65 | 0.27 ± 0.00 |  | *Carex acutiformis* |
|  | H47 | 0.26 ± 0.00 | 44.0 ± 0.10 | *Carex acutiformis* |
|  | L51 | 0.31 ± 0.00 | 43.4 ± 0.11 | *Carex acutiformis* |
|  | O66 | 0.26 ± 0.00 | 43.3 ± 0.30 | *Carex acutiformis* |
|  | U96 | 0.40 ± 0.00 | 44.7 ± 0.10 | *Carex acutiformis* |
|  | X16 | 0.29 ± 0.00 | 43.5 ± 0.06 | *Carex acutiformis* |
| *S. fluviatilis* | I68 | 0.86 ± 0.01 | 39.3 ± 0.12 | *Solanum pseudocapsicum* |
|  | J53 | 0.90 ± 0.01 | 39.7 ± 0.09 | *Solanum pseudocapsicum* |
|  | J87 | 0.90 ± 0.01 | 38.7 ± 0.13 | *Solanum pseudocapsicum* |
| *S. hibernica* | 105.F6 | 1.82 ± 0.02 | 39.5 ± 0.14 | *Solanum pseudocapsicum* |
|  | I54 | 1.81 ± 0.01 |  | *Solanum pseudocapsicum* |
|  | I81 | 1.79 ± 0.02 |  | *Solanum pseudocapsicum* |
|  | I89 | 1.81 ± 0.03 | 40.1 ± 0.23 | *Solanum pseudocapsicum* |
|  | J84 | 1.78 ± 0.04 |  | *Solanum pseudocapsicum* |
|  | J88 | 1.77 ± 0.03 | 39.5 ± 0.10 | *Solanum pseudocapsicum* |
|  | S103.D5 | 1.84 ± 0.01 |  | *Solanum pseudocapsicum* |
|  | SIE104_D11 | 1.87 ± 0.02 |  | *Solanum pseudocapsicum* |
|  | X76 | 1.68 ± 0.01 | 40.0 ± 0.23 | *Solanum pseudocapsicum* |
| *S. lanceolata* | H88 | 0.99 ± 0.01 | 39.1 ± 0.16 | *Solanum pseudocapsicum* |
|  | S89.G5 | 1.04 ± 0.01 | 39.9 ± 0.15 | *Solanum pseudocapsicum* |
| *S. laticarina* | R93 | 1.91 ± 0.03 |  | *Solanum pseudocapsicum* |
|  | S90.C8 | 1.81 ± 0.03 | 42.1 ± 0.07 | *Solanum pseudocapsicum* |
|  | T80 | 1.80 ± 0.01 |  | *Solanum pseudocapsicum* |
|  | U17 | 1.78 ± 0.00 | 41.7 ± 0.37 | *Solanum pseudocapsicum* |
|  | U93 | 1.85 ± 0.02 |  | *Solanum pseudocapsicum* |
| *S. leptorrhabda* | H92 | 0.19 ± 0.00 | 41.3 ± 0.17 | *Carex acutiformis* |
|  | I41 | 0.21 ± 0.00 | 42.3 ± 0.18 | *Carex acutiformis* |
|  | I13 | 0.21 ± 0.00 | 41.7 ± 0.20 | *Carex acutiformis* |
|  | J50 | 0.28 ± 0.00 |  | *Carex acutiformis* |
|  | SIE105A | 0.47 ± 0.00 | 42.1 ± 0.11 | *Solanum pseudocapsicum* |
|  | U73 | 0.34 ± 0.00 | 40.7 ± 0.10 | *Carex acutiformis* |
| *S. praefracta* | I32 | 1.08 ± 0.02 | 39.8 ± 0.13 | *Solanum pseudocapsicum* |
| *S. rubra* | C74 | 1.10 ± 0.02 |  | *Solanum pseudocapsicum* |
|  | NIES 695 | 0.81 ± 0.01 | 45.2 ± 0.20 | *Solanum pseudocapsicum* |
| *S. sp.* | E22 | 1.06 ± 0.03 |  | *Solanum pseudocapsicum* |
|  | L65 | 0.9 ± 0.01 | 39.4 ± 0.10 | *Solanum pseudocapsicum* |
|  | M24 | 0.98 ± 0.01 | 40.7 ± 0.13 | *Solanum pseudocapsicum* |
|  | S113.E3 | 2.41 ± 0.03 | 40.6 ± 0.08 | *Bellis perennis* |
|  | S54.E11 | 1.46 ± 0.02 | 41.4 ± 0.20 | *Solanum pseudocapsicum* |
|  | T35 | 1.09 ± 0.01 | 39.8 ± 0.28 | *Solanum pseudocapsicum* |
|  | T83 | 2.15 ± 0.02 | 40.8 ± 0.15 | *Solanum pseudocapsicum* |
|  | U20 | 2.22 ± 0.02 | 40.5 ± 0.25 | *Solanum pseudocapsicum* |
|  | U66 | 2.27 ± 0.01 | 40.8 ± 0.25 | *Solanum pseudocapsicum* |
|  | V17 | 2.21 ± 0.02 |  | *Solanum pseudocapsicum* |
|  | V41 | 1.96 ± 0.03 | 38.0 ± 0.40 | *Solanum pseudocapsicum* |
|  | X28 | 3.51 ± 0.04 | 41.1 ± 0.21 | *Solanum pseudocapsicum* |
|  | X31 | 1.61 ± 0.01 | 38.4 ± 0.07 | *Solanum pseudocapsicum* |
|  | X37 | 2.25 ± 0.03 | 40.8 ± 0.28 | *Solanum pseudocapsicum* |
|  | X63 | 1.46 ± 0.02 | 39.4 ± 0.28 | *Solanum pseudocapsicum* |
| *S. spinosa* | CZ10D | 1.23 |  | *Carex acutiformis* |
|  | S117.C6 | 2.22 ± 0.01 | 41.7 ± 0.25 | *Bellis perennis* |
| *S. splendida* | T2 | 1.11 ± 0.02 |  | *Solanum pseudocapsicum* |
| *S. synuroidea* | S95E5 | 0.30 ± 0.00 | 43.7 ± 0.16 | *Carex acutiformis* |
| *S. truttae* | E29 | 1.50 ± 0.02 |  | *Solanum pseudocapsicum* |
|  | I20 | 1.53 ± 0.02 |  | *Solanum pseudocapsicum* |
|  | I30 | 1.54 ± 0.02 | 40.8 ± 0.23 | *Solanum pseudocapsicum* |
|  | Q6 | 1.02 ± 0.01 | 39.9 ± 0.09 | *Solanum pseudocapsicum* |
|  | S34.1 | 1.10 ± 0.01 | 40.2 ± 0.04 | *Solanum pseudocapsicum* |
|  | T61 | 1.47 ± 0.01 | 39.6 ± 0.62 | *Solanum pseudocapsicum* |
| *S. uvella* | L64 | 0.80 ± 0.01 | 37.7 ± 0.05 | *Solanum pseudocapsicum* |
| *S. vinlandica* | I82 | 1.14 ± 0.02 | 39.3 ± 0.13 | *Solanum pseudocapsicum* |
